# Genetic Factors of Elite Wrestling Status: A Multi-Ethnic Comparative Study

**DOI:** 10.3390/genes16080906

**Published:** 2025-07-29

**Authors:** Ayumu Kozuma, Celal Bulgay, Hirofumi Zempo, Mika Saito, Minoru Deguchi, Hiroki Homma, Shingo Matsumoto, Ryutaro Matsumoto, Anıl Kasakolu, Hasan H. Kazan, Türker Bıyıklı, Seyrani Koncagül, Giyasettin Baydaş, Mehmet A. Ergun, Attila Szabo, Ekaterina A. Semenova, Andrey K. Larin, Nikolay A. Kulemin, Edward V. Generozov, Takanobu Okamoto, Koichi Nakazato, Ildus I. Ahmetov, Naoki Kikuchi

**Affiliations:** 1 Graduate School of Health and Sport Science, Nippon Sport Science University, Tokyo 158-8508, Japan; 23sda08@nittai.ac.jp (A.K.); sa123ka.v@gmail.com (M.S.); hiroki0145@gmail.com (H.H.); matsumoto-s@nittai.ac.jp (S.M.); tokamoto@nittai.ac.jp (T.O.); nakazato@nittai.ac.jp (K.N.); 2 Sports Science Faculty, Bingöl University, Bingöl 12000, Türkiye; cbulgay@bingol.edu.tr; 3 Faculty of Health and Nutrition, Tokyo Seiei College, Tokyo 124-8530, Japan; zempo.hirofumi@gmail.com; 4 Faculty of Health Sciences, Kyorin University, Tokyo 192-8508, Japan; minoru-deguchi@ks.kyorin-u.ac.jp; 5 Faculty of Education, Ikuei University, Gumma 370-0011, Japan; rmatsumoto@ikuei-g.ac.jp; 6 Graduate School of Natural and Applied Sciences, Ankara University, Ankara 06135, Türkiye; akasakolu@ankara.edu.tr; 7 Department of Medical Biology, Gulhane Faculty of Medicine, University of Health Sciences, Ankara 06010, Türkiye; hasanhuseyin.kazan@sbu.edu.tr; 8 Sports Science Faculty, Marmara University, İstanbul 34722, Türkiye; turkerbiyikli@marmara.edu.tr; 9 Department of Animal Science, Faculty of Agriculture, Ankara University, Ankara 06135, Türkiye; koncagul@ankara.edu.tr; 10 Department of Physiology, Faculty of Medicine, İstanbul Medeniyet University, İstanbul 34700, Türkiye; giyasettin.baydas@medeniyet.edu.tr; 11 Department of Medical Genetics, Faculty of Medicine, Gaz University, Ankara 06560, Türkiye; aliergun@gazi.edu.tr; 12 Faculty of Health and Sport Sciences, Széchenyi István University, H-9026 Győr, Hungary; szabo.attila@sze.hu; 13 Department of Molecular Biology and Genetics, Lopukhin Federal Research and Clinical Center of Physical-Chemical Medicine of Federal Medical Biological Agency, 119435 Moscow, Russia; alecsekaterina@gmail.com (E.A.S.); zelaz@yandex.ru (A.K.L.); maveriksvao@gmail.com (N.A.K.); generozov@gmail.com (E.V.G.); i.akhmetov@ljmu.ac.uk (I.I.A.); 14 Research Institute of Physical Culture and Sport, Volga Region State University of Physical Culture, Sport and Tourism, 420138 Kazan, Russia; 15 Laboratory of Genetics of Aging and Longevity, Kazan State Medical University, 420012 Kazan, Russia; 16 Research Institute for Sport and Exercise Sciences, Liverpool John Moores University, Liverpool L3 5AF, UK

**Keywords:** genome-wide association study, gene expression, athlete status, comprehensive analysis, genotype, talent, skeletal muscle

## Abstract

Background: In recent years, comprehensive analyses using a genome-wide association study (GWAS) have been conducted to identify genetic factors related to athletic performance. In this study, we investigated the association between genetic variants and elite wrestling status across multiple ethnic groups using a genome-wide genotyping approach. Methods: This study included 168 elite wrestlers (64 Japanese, 67 Turkish, and 36 Russian), all of whom had competed in international tournaments, including the Olympic Games. Control groups consisted of 306 Japanese, 137 Turkish, and 173 Russian individuals without elite athletic backgrounds. We performed a GWAS comparing allele frequencies of single-nucleotide polymorphisms (SNPs) between elite wrestlers and controls in each ethnic cohort. Cross-population analysis comprised (1) identifying SNPs with nominal significance (*p* < 0.05) in all three groups, then (2) meta-analyzing overlapped SNPs to assess effect consistency and combined significance. Finally, we investigated whether the most significant SNPs were associated with gene expression in skeletal muscle in 23 physically active men. Results: The GWAS identified 328,388 (Japanese), 23,932 (Turkish), and 30,385 (Russian) SNPs reaching nominal significance. Meta-analysis revealed that the *ATP2A3* rs6502758 and *UNC5C* rs265061 polymorphisms were associated (*p* < 0.0001) with elite wrestling status across all three populations. Both variants are located in intronic regions and influence the expression of their respective genes in skeletal muscle. Conclusions: This is the first study to investigate gene polymorphisms associated with elite wrestling status in a multi-ethnic cohort. *ATP2A3* rs6502758 and *UNC5C* rs265061 polymorphisms may represent important genetic factors associated with achieving an elite status in wrestling, irrespective of ethnicity.

## 1. Introduction

Wrestling is a combat sport that is popular among various ethnic groups. It is also one of the sports events featured in the Olympic Games, included since 1896 for male athletes (except in 1900) and since 2004 for female athletes. Success in wrestling requires a high level of athletic performance. Previous studies have reported that international-level wrestlers have greater muscle strength, power, and anaerobic capacity than national-level wrestlers [1,2,3]. These differences have also been observed within the international level, where medalists show a higher anaerobic capacity, strength, power, and faster reaction times than non-medalists [4,5]. Anaerobic capacity, in particular, is considered a key factor for success regardless of age category, weight class, and wrestling style. Moreover, muscle strength and power are also essential components for achieving success in wrestling [6].

Genetic factors are thought to contribute to these abilities. Although genetic studies on wrestlers remain limited, several studies have reported associations with wrestling status. For example, the *ACTN3* R577X polymorphism, which affects the production of α-actinin-3, a structural protein in fast-twitch muscle fibers [7,8,9], and the *ACE* I/D polymorphism, which is involved in the renin–angiotensin system and influences ACE enzyme activity in serum and muscle [7,10,11,12], have been linked to wrestling performance. In addition, other polymorphisms, such as *GALNTL6* rs558129 [13], and *HIF1A* Pro582Ser [14], have also been reported. These polymorphisms are related to physical fitness components, such as muscle strength, power, anaerobic capacity, and reaction time, and have been associated with wrestling status. Therefore, further investigation of these genetic factors in wrestlers may lead to practical applications in sports science and athlete development. Therefore, further investigation of these genetic factors in wrestlers may lead to practical applications in the sports field.

Genetic studies involving elite athletes have long faced challenges in securing sufficient sample sizes due to the limited population. Therefore, studies including multiple countries may be essential [15]. In recent years, international initiatives, such as the Athlome Project Consortium, have advanced the exploration of gene polymorphisms related to elite athletes’ performance [16,17]. In particular, advancements in genotyping technologies have enabled genome-wide association study (GWAS) to be used for comprehensive analyses. This approach allows for the comprehensive analysis of hundreds of thousands of gene polymorphisms, making it possible to identify novel associated gene polymorphisms for specific traits.

Several GWASs have been conducted on athletes [18,19,20,21,22,23], with many including participants from diverse sports. For example, Al-Khelaifi et al. [20] analyzed athletes across 29 different sports in their discovery phase, followed by a replication phase involving endurance athletes (17 sports) and power/strength-oriented athletes (14 sports). However, the research focusing on athletes from a single sport remains limited. Additionally, few studies have explored gene polymorphisms associated with elite athletic performance across different ethnic groups.

Given these gaps, this study aimed to investigate the association between genetic variants and elite wrestling status using genome-wide genotyping across multiple countries (Japan, Türkiye, and Russia).

## 2. Materials and Methods

### 2.1. The Ethics Statement

The Ethics Committees of the Nippon Sport Science University (Approval number: 020-G03), the Clinical Research Ethics Committee of Gazi University (Approval No: 2023/642), and the Federal Research and Clinical Center of Physical–Chemical Medicine of the Federal Medical and Biological Agency of Russia (Approval number 2017/04) approved the protocols for this research. Both verbal and written informed consent were obtained from all participants involved in this study. This study was conducted according to the guidelines of the Declaration of Helsinki and Strengthening The Reporting of Genetic Association Studies (STREGA): An extension of the Strengthening the Reporting of Observational Studies in Epidemiology (STROBE) statement recommendations.

### 2.2. Participants

This study included participants from three countries. Specifically, 64 elite wrestlers (54 male and 10 female; mean age: 24.9 ± 8.8 years) and 306 controls (143 male and 163 female; mean age: 68.5 ± 12.7 years) from Japan, elite 67 male wrestlers (mean age: 27.8 ± 3.7 years) and 137 male controls (mean age: 42.3 ± 4.9 years) from Türkiye, and highly elite 36 wrestlers (15 male and 21 female; mean age: 30.1 ± 3.6 years) and 173 controls (137 male and 36 female; mean age: 44.5 ± 4.1 years) from Russia were included. The controls were healthy individuals, and the competition level of the wrestlers who participated in the international competitions, including the Olympic Games, was included. All Russian wrestlers were medalists in international competitions. The level of competition was confirmed by results using official results and questionnaires. The gene expression study analyzed vastus lateralis muscle samples from the independent cohort of 23 physically active Russian men of European descent, including 10 power-trained athletes (mean ± SD: age: 30.1 ± 7.4 years, height: 178.2 ± 6.7 cm, and body mass: 85.6 ± 12.4 kg) and 13 endurance-trained athletes (age: 34.2 ± 10.0 years, height: 182.8 ± 6.8 cm, and body mass: 76.0 ± 9.9 kg).

### 2.3. Genotyping

#### 2.3.1. Japanese Cohorts

Saliva samples were collected using an Oragene DNA Kit (DNA Genotek, Ottawa, ON, Canada). Saliva samples were incubated at 50 °C for at least 1 h to ensure complete cell lysis. Subsequently, a purification reagent (PT-L2P) was added to precipitate proteins and other impurities. The mixture was centrifuged, and the supernatant containing DNA was carefully transferred to a new tube. DNA was then precipitated using ethanol, followed by washing to remove residual impurities. Finally, the DNA pellet was resuspended in TE buffer or an appropriate elution buffer and stored at 4 °C until further analysis.

The total DNA samples were genotyped using the Japonica SNP array (Toshiba Co., Tokyo, Japan). Genotype calls were performed using Axiom Analysis Suite (version 5.1.1., Thermo Fisher Scientific, Waltham, MA, USA). For quality control (QC), single nucleotide polymorphisms (SNPs) were excluded based on the following criteria: a missing rate of >1%, minor allele frequency (MAF) < 0.005, and Hardy–Weinberg equilibrium (HWE) *p*-value < 1 × 10^−4^. Relatedness among samples was estimated based on identity-by-state (IBS) and identity-by-descent (IBD) factors, and PI_HAT values were calculated. For each pair of individuals with a PI_HAT value greater than 0.1 (indicating up to third-degree relatedness), one individual was removed. After QC, a total of 627,920 SNPs remained.

For imputation, we performed SHAPEIT v2 and IMPUTE2 v2 on the 3.5 K Japanese reference panel (developed by Tohoku Medical Megabank Organization). Post-imputation QC criteria included an imputation quality measure > 0.7, a MAF < 0.01, genotype missingness > 0.03, sample missingness > 0.1, and HWE *p* value < 1 × 10^−6^. A total of 7,463,336 variants remained and were used for the GWAS.

#### 2.3.2. Russian Cohorts

DNA was extracted from leukocytes obtained from venous blood samples (4 mL) collected in EDTA tubes (Vacuette EDTA tubes, Greiner Bio-One, Kremsmünster, Austria). Samples were kept at 4 °C during transport and DNA was extracted on the same day using a commercial kit (Technoclon, Moscow, Russia) following the manufacturer’s protocol. The procedure involved chemical lysis, selective DNA binding on silica spin columns, and ethanol washing. DNA quality was confirmed by agarose gel electrophoresis.

Genotyping was performed using HumanOmni1-Quad BeadChips (Illumina Inc., San Diego, CA, USA), covering 1,140,419 SNPs. The assay required 200 ng of DNA at a minimum concentration of 50 ng/µL. DNA concentrations were measured using a Qubit Fluorometer (Invitrogen, Waltham, MA, USA). All downstream steps followed the Infinium HD Assay protocol. For QC, SNPs with a MAF below 0.05, a missing rate exceeding 0.05, or a HWE *p* value less than 0.00001 were excluded. No imputation was performed for this cohort.

#### 2.3.3. Turkish Cohorts

Peripheric venous blood samples of 4 mL in an EDTA-containing collection tube for each participant were used for DNA extraction. Genomic DNAs from blood was isolated using DNeasy Blood and Tissue Kits (Qiagen, AG, Basel, Switzerland) according to the routine protocol of the supplier. DNA concentration was determined using NanoDrop 1000 (Thermo Scientific, Waltham, MA, USA). DNA integrity was assessed by running on a 1% agarose gel. Isolated DNAs were stored at −20 °C for long-term storage.

The total DNA samples were genotyped using an Infinium Global Screening Array-24 + v3.0 Booster chip (Illumina Inc., San Diego, CA, USA) using the routine protocol of the supplier. The genotypes were called using GenomeStudio Software 2.0.5 (Illumina Inc., San Diego, CA, USA) covering 604,625 variants. QC procedures excluded SNPs on sex chromosomes, those with a missing genotype rate exceeding 0.1, a MAF below 0.01, or a HWE *p* value less than 1 × 10^−13^. Following QC filtering, 425,254 SNPs were retained for analysis. No imputation was performed for this cohort.

### 2.4. Gene Expression Analysis

Transcriptomic analysis was conducted using RNA-seq in 23 physically active men. The sequencing data were deposited in the Gene Expression Omnibus (GEO) under accession number GSE200398. To minimize confounding effects from recent exercise, participants abstained from training for ≥24 h before muscle biopsy (collected using the Bergström needle technique). Muscle-derived RNA was isolated using the RNeasy Mini Fibrous Tissue Kit (Qiagen, Hilden, Germany). Concentration was quantified via Qubit spectrophotometry (Thermo Fisher Scientific, USA), and integrity was assessed using the BioAnalyzer 2100 system with the RNA Nano assay (Agilent Technologies, Santa Clara, CA, USA). Only samples with RNA integrity numbers (RINs) > 7 were retained. DNase I-treated RNA (Turbo DNA-free Kit, Thermo Fisher Scientific) was used to prepare sequencing libraries with the NEBNext Ultra II Directional RNA Library Prep Kit and rRNA Depletion Module (New England Biolabs, Ipswich, MA, USA). Libraries were sequenced on an Illumina HiSeq platform (250 cycles). Gene-level expression was quantified in transcripts per kilobase million (TPM), with focused analysis on *ATP2A3* and *UNC5C* expression patterns. For the whole transcriptome analysis, quality control by FastQC [24] and MultiQC [25] was performed before and after adapter trimming by Cutadapt (version 3.3) [26] and quality filtering by trimmomatic (version 0.39) [27] for the whole dataset. The method of the trimmed mean of M-values (TMM) was applied for the normalization of the library sizes. TMM-normalized counts per million (CPM) data from edgeR were used through filtering by maximal mean in any group > 10 CPM.

### 2.5. Statistical Analysis

PLINK1.9 or 2.0 (Bethesda, MD, USA) was used for quality control checks [28,29]. In the Japanese and Russian cohorts, GWASs were performed under an additive model. In the Turkish cohort, associations were examined using Fisher’s exact test. SNPs that reached a nominal significance level of *p* < 0.05 in all three cohorts were selected as candidates for further analysis. This approach follows previous studies that used liberal thresholds at the discovery stage to avoid missing potentially relevant associations [19]. Hardy–Weinberg equilibrium (HWE) was assessed using Pearson’s chi-squared test, but only for the SNPs that showed an association in all three countries. Although the statistical approaches for the GWAS differed between cohorts, allele frequencies were extracted from all cohorts and a meta-analysis was conducted using the Review Manager software program (version 5.3; Copenhagen: The Nordic Cochrane Center, The Cochrane Collaboration, http://tech.cochrane.org/revman accessed on 16 June 2025). A random effect model was applied. Heterogeneity across cohorts was evaluated using the mean of I^2^ statistics. Relationships between gene polymorphisms and the expression of corresponding genes were assessed using regression analysis adjusted for covariates (age, type of training (power/endurance), and training frequency). Statistical significance was set at *p* < 0.05.

## 3. Results

### 3.1. Case–Control Studies

**Figure 1:**
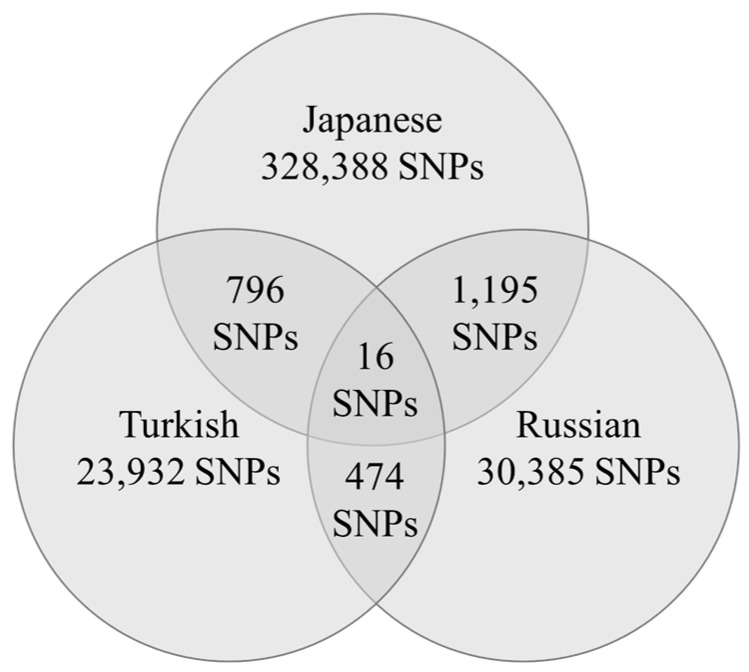
Number of gene polymorphisms that reached the significance level in each country and the number of gene polymorphisms in common.

The number of gene polymorphisms that reached the nominal statistical significance was 328,388 in Japanese participants, 23,932 in Turkish participants, and 30,385 in Russian participants (Figure 1).

Sixteen gene polymorphisms were associated with wrestler status across all three populations (Table 1 and Figure 1). HWE tests were performed for each of the sixteen polymorphisms. Among Japanese wrestlers, only the rs774294 polymorphism showed a deviation from HWE (*p* = 0.02), while all other polymorphisms conformed to HWE across all three countries.

**Figure 2:**
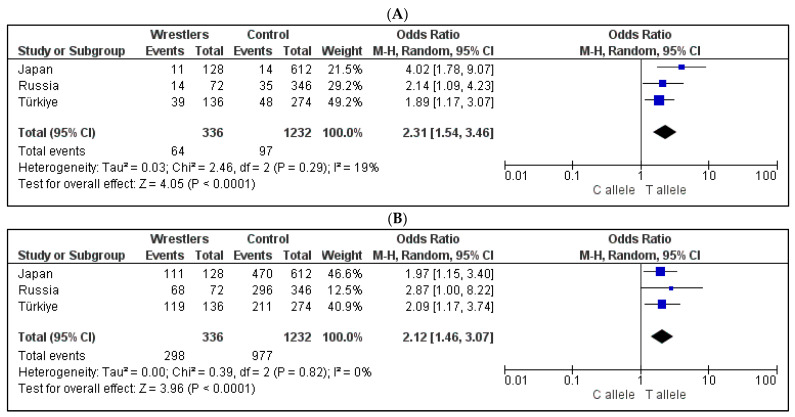
The results of meta-analyses of three countries for the association between the (**A**) *ATP2A3* rs6502758 polymorphism and (**B**) *UNC5C* rs265061 polymorphism.

The meta-analysis showed that the *ATP2A3* rs6502758 T and *UNC5C* rs265061 T alleles were significantly associated with wrestler status in the same direction across all three countries (Figure 2, *p* < 0.0001). The minor allele (T) frequency of *ATP2A3* rs6502758 was 2.3% in Japanese controls and 8.6% in Japanese wrestlers, 17.5% in Turkish controls and 31.4% in Turkish wrestlers, and 10.1% in Russian controls and 19.4% in Russian wrestlers. The minor allele (C) frequency of *UNC5C* rs265061 was 23.2% in Japanese controls and 13.3% in Japanese wrestlers, 23.0% in Turkish controls and 12.5% in Turkish wrestlers, and 36.2% in Russian controls and 5.6% in Russian wrestlers.

### 3.2. Gene Expression Study

**Figure 3:**
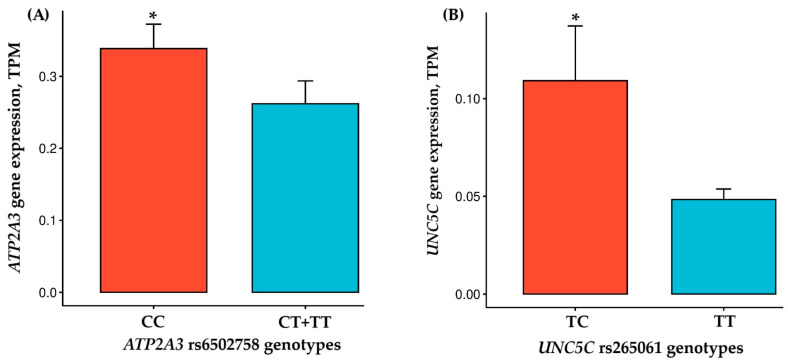
Comparison of *ATP2A3* (**A**) and *UNC5C* (**B**) gene expressions between individuals with different genotypes. * *p* < 0.05, statistically significant differences adjusted for covariates. TPM, transcripts per kilobase million.

The genotype distributions for *ATP2A3* rs6502758 (CC = 14, CT = 8, TT = 1; HWE χ^2^ = 0.011, *p* = 0.915) and *UNC5C* rs265061 (TT = 21, TC = 2, CC = 0; χ^2^ = 0.047, *p* = 0.827) were in Hardy–Weinberg equilibrium among the 23 male participants. No significant allele frequency differences were observed between power-trained and endurance-trained groups for either *ATP2A3* rs6502758 (CC = 8, CT = 1, TT = 1 vs. CC = 6, CT = 7, TT = 0; *p* > 0.05) or *UNC5C* rs265061 (TT = 9, TC = 1, CC = 0 vs. TT = 12, TC = 1, CC = 0; *p* > 0.05), potentially reflecting limited statistical power in our modest sample size. Analysis of vastus lateralis muscle samples from 23 physically active men revealed a significantly higher *ATP2A3* expression in rs6502758 CC genotype carriers compared to T allele carriers (0.34 ± 0.13 vs. 0.26 ± 0.09 TPM; covariate-adjusted *p* = 0.0042). Additionally, *UNC5C* expression was significantly elevated in rs265061 TC genotype carriers relative to TT homozygotes (0.11 ± 0.04 vs. 0.05 ± 0.02 TPM; covariate-adjusted *p* = 0.024) (Figure 3). No significant differences in gene expression were observed between power-trained and endurance-trained subjects for *ATP2A3* (0.30 ± 0.17 vs. 0.35 ± 0.12 TPM, *p* = 0.430) or *UNC5C* (0.058 ± 0.04 vs. 0.051 ± 0.02 TPM, *p* = 0.582).

## 4. Discussion

This study aimed to examine the association between genetic variants and elite wrestling performance across multiple nationalities (Japan, Türkiye, and Russia) through genome-wide genotyping. Sixteen gene polymorphisms demonstrated statistical significance across all three populations, with two variants (*ATP2A3* rs6502758 and *UNC5C* rs265061) showing concordant effect directions in their associations. Notably, functional characterization revealed these intronic variants significantly modulate the expression of their respective genes in skeletal muscle tissue. Several gene polymorphisms associated with wrestling status have been previously reported [7,13,14]. In this study, however, only the *ACE* gene rs4341 polymorphism showed a significant association, and this was observed exclusively in the Turkish cohort (*p* = 0.008). No associations were found for other previously reported SNPs across the three populations. Therefore, the identification of two novel polymorphisms—*ATP2A3* gene rs6502758 and *UNC5C* gene rs265061—with consistent associations across multiple ethnic groups is particularly noteworthy.

The Unc-5 netrin receptor C (*UNC5C*) gene, which belongs to the UNC-5 family of netrin receptors and functions as a receptor for netrin-1, is located on chromosome 4. Netrin-1 has been found to be an important factor in embryonic development, with functions in axon guidance, cell migration, morphogenesis, and angiogenesis [30]. Previous studies using Unc5c knockout mice have reported that Unc5c is widely expressed in neurons in the precerebellar and deep cerebellar neurons, leading to neurodevelopmental defects, such as cerebellar hypoplasia and ataxia [31]. Unc5c has also been shown to play a role in axon guidance and the pathway selection of nerve growth cones through complex signaling with Netrin-1 and ephrinB2 during neuronal circuit formation [32]. A recent study reported that the NTN1/DCC signaling pathway involving UNC5C plays a critical role in axon guidance [33]. In addition, mutations in the *UNC5C* gene have been shown to disrupt the cytoplasmic functional ZU5 domain and the region required for netrin-mediated axon repulsion. Although an association between *UNC5C* gene rs265061 polymorphism and athletic performance has not been established, its role in neuronal development may affect wrestling performance.

The ATPase sarcoplasmic/endoplasmic reticulum Ca2+ transporting 3 (*ATP2A3*) gene is located on chromosome 17 and encodes one of the Sarco/Endoplasmic Reticulum Calcium ATPases (SERCAs). SERCA transports Ca2+ from the cytosol to the endoplasmic reticulum and plays an important role in intracellular calcium homeostasis, with three major isoforms: SERCA1, SERCA2, and SERCA3 [34]. SERCA1 is expressed in fast-twitch muscle fibers, while SERCA2 is expressed in heart and slow-twitch muscle fibers. SERCA3 has been reported to be expressed mainly in non-muscle cells, with multiple selectively spliced isoforms, suggesting that its diversity is complex [34]. Although SERCA3 expression is very rare in muscle tissue, its overall structure is highly conserved, with approximately 75% similarity to SERCA1 [35]. Therefore, SERCA3 is predicted to exhibit similar sensitivity to Ca^2+^ and enzyme activity. In addition, SERCA3 has been reported to be involved in the regulation of relaxation of vascular and tracheal smooth muscle. These findings suggest that, despite its limited role in skeletal muscle, ATP2A3 may contribute to calcium handling. Interestingly, the T allele of rs6502758 has been associated with a decreased risk of shoulder and upper arm injuries in the FinnGen cohort (*p* = 0.0045; *n* = 241,940). Given that wrestling demands the repetitive and intense use of the upper limbs for techniques such as throws and movement on the ground, a genetic factor to reduce injury risk in these regions could support performance longevity and training continuity. Therefore, this polymorphism may have supported the musculoskeletal resilience required for high-level wrestling performance.

Although this study underlined novel variants associated with wrestling through different populations, there were several limitations. First, despite combining three populations, the overall sample size remained limited because this study included only elite wrestlers, which raises concerns about statistical power and the reproducibility of the findings. Moreover, SNP arrays, genotype calls, and statistical methods used for GWASs differed among the three countries. These differences may have introduced technical variability. Therefore, future studies should be conducted using standardized methods. This study is limited to a comparison across three countries. In the future, conducting research on more diverse populations is expected to deepen our understanding of the genetic characteristics of wrestlers. There was also a notable age difference between the Japanese wrestlers and controls. However, the allele frequencies of the controls were similar to those reported in a public database of the general Japanese population (jMorp), suggesting that this age difference did not substantially bias the genetic background of the controls. Finally, while some gene polymorphisms showed consistent effect directions across the three countries, the magnitude of the odds ratios and allele frequencies varied by ethnicity. These differences may reflect ethnic-specific genetic backgrounds.

## 5. Conclusions

In conclusion, the results of this study show that the *ATP2A3* gene rs6502758 polymorphism and *UNC5C* gene rs265061 polymorphism were consistently associated with elite wrestling performance across all three populations. These gene polymorphisms might play a significant role in elite wrestling performance, regardless of nationality. Future studies may focus on exploring the functional roles of these gene polymorphisms. The application of a polygenic risk score could help to better understand how genetic factors contribute to athletic characteristics. In addition, although this study focused on gene polymorphisms, athlete status is influenced by a complex interplay of genetic and environmental factors. Future studies should aim to explore these potential gene–environment interactions to obtain a more comprehensive understanding of athletic traits. Furthermore, examinations of other ethnic groups, other types of athletic competitions, and large cohorts are expected to enhance the reproducibility and applicability of our findings.

## Figures and Tables

**Table 1:** Meta-analysis of 16 SNPs with shared nominal significance identified in genetic polymorphism studies of elite wrestlers from three countries.

				Japan		Russia		Türkiye		Meta-Analysis		
rs Number	Chr	Gene Name	Effect Allele	OR	*p*	OR	*p*	OR	*p*	OR	*p*	I^2^
rs4655854	1	-	A	0.64	0.023	1.68	0.044	0.48	0.003	0.80	0.52	85
rs12479172	2	-	T	1.60	0.025	1.96	0.011	0.63	0.032	1.24	0.53	86
rs7698692	4	-	A	1.48	0.045	2.46	0.030	0.36	0.028	1.14	0.78	81
rs12505795	4		T	1.84	0.007	2.27	0.038	0.38	0.046	1.25	0.64	79
rs265061	4	*UNC5C*	T	1.97	0.016	2.87	0.041	2.09	0.011	2.12	<0.0001	0
rs202821	5	-	A	2.05	0.011	8.17	0.047	0.62	0.036	2.15	0.31	96
rs3789243	7	*ABCB1*	A	0.66	0.049	1.94	0.011	0.55	0.006	0.87	0.71	87
rs11203550	8	-	A	2.57	0.0009	0.20	0.003	2.05	0.043	1.14	0.83	88
rs4131754	8	-	T	1.52	0.032	2.15	0.012	0.64	0.048	1.25	0.52	84
rs10893471	11	*CNTN5*	A	0.59	0.043	0.60	0.047	1.36	0.003	0.79	0.41	74
rs9988991	12	*GRIN2B*	A	1.77	0.025	0.56	0.028	1.45	0.042	1.13	0.71	82
rs7170004	15	*IQCH*	A	2.90	0.006	0.58	0.039	1.59	0.035	1.36	0.48	87
rs2732158	15	*SH3GL3*	T	0.50	0.039	2.64	0.004	0.45	0.045	0.84	0.75	88
rs7187994	16	*USP10*	A	0.58	0.003	2.15	0.032	1.85	0.013	1.28	0.59	89
rs774294	16	*USP10*	A	0.58	0.006	1.70	0.042	2.71	3.85 × 10^−6^	1.38	0.51	93
rs6502758	17	*ATP2A3*	T	4.02	0.001	2.14	0.025	1.89	0.013	2.31	<0.0001	19

## Data Availability

Total RNA sequencing data are deposited in GEO, accession number GSE200398. The GWAS data presented in this study are available on request from the second author and corresponding author.
